# Regulation of Plant Growth and Development by Melatonin

**DOI:** 10.3390/life14121606

**Published:** 2024-12-04

**Authors:** Dawei Shi, Lejia Zhao, Ruijia Zhang, Qiaofeng Song

**Affiliations:** 1 Co-Innovation Center for Sustainable Forestry in Southern China, College of Life Sciences, Nanjing Forestry University, 159 Long pan Road, Nanjing 210037, China; 2 College of Forestry, Nanjing Forestry University, 159 Long pan Road, Nanjing 210037, China; zhaolejia2003@njfu.edu.cn (L.Z.); viiridis@njfu.edu.cn (R.Z.); 3 Human Phenome Institute (HuPI), Fudan University, 825 Zhangheng Road, Pudong District, Shanghai 200100, China; qfsong23@m.fudan.edu.cn

**Keywords:** melatonin, plant hormone, growth, reproduction, plant stress, signaling pathways

## Abstract

Melatonin is a naturally occurring chemical with pleiotropic effects in various species. In plants, melatonin is associated with a variety of plant physiological processes, including plant growth and development, stress responses, etc. Thus, melatonin may hold promise for improving crop yields and agricultural sustainability. This review describes the biosynthetic mode of melatonin and its properties and summarizes its functions in growth, development, and reproduction. In addition, the role of melatonin in plants facing various stressful environments is elaborated upon, and its relationship with other phytohormones is summarized. Through this review, we recognize the problems and challenges facing melatonin research and propose some feasible solutions.

## 1. Introduction

Melatonin has been classified as an animal hormone since its discovery as a small-molecule growth regulator in the bovine pineal gland in 1958 [1]. It did not receive attention from the plant scientific community until its discovery in *Pharbitis nil* as well as the discovery of its related growth regulators [2]. Melatonin is widely present in higher plants and can regulate circadian and photoperiod responses by scavenging reactive oxygen species (ROS) to protect leaves from peroxide effect [3]. In 2018, Arabidopsis cell membrane melatonin receptors were discovered, and the status of melatonin as a novel phytohormone gradually started to be recognized by the academic community [4].

In animals, melatonin regulates the circadian cycle, mood, sleep, body temperature, and immune function [3,5,6]. Recent research has discovered that melatonin, a hormone that was initially known for its regulatory functions in mammalian circadian rhythms, has a broad range of functions in plants as well. Notably, melatonin is linked to fruit development and the regulation of plant senescence progression. It is also involved in various physiological processes, such as leaf growth, reproduction, and stress response. A large number of studies have confirmed that almost all plants and even related products contain melatonin, but the content varies greatly according to different species, parts of the plant itself, developmental stages, and external environments.

In this review, we present the current knowledge on melatonin’s biosynthesis, actions, and synthetic mechanisms; its role in plants; and new research developments in melatonin signal transduction pathways, and we assess its future applicability in agricultural production.

## 2. Melatonin Biosynthesis

**Figure 1:**
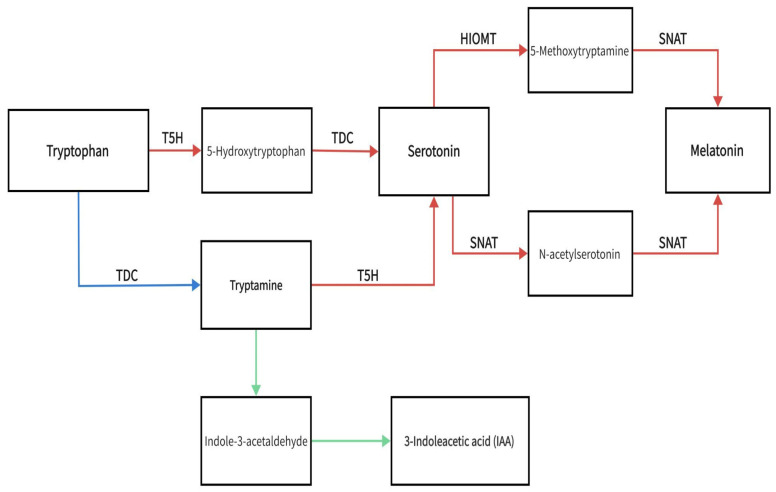
Melatonin and 3-indoleacetic acid (IAA) biosynthetic pathway.

The melatonin precursor molecule tryptophan is converted to 5-hydroxytryptophan in the vertebrate pineal gland via catalysis by tryptophan-5-hydroxylase, which is the primary mechanism of melatonin generation in the pineal gland [7]. This tryptophan route was later identified in plants, in which 5-hydroxytryptophan is transformed into serotonin, which is then turned into N-acetylserotonin via catalysis by serotonin N-acetyltransferase (SNAT). Eventually, oxindole-o-methyltransferase/acetylserotonin methyltransferase (HIOMT/ASMT) catalyzes the transformation of N-acetylserotonin to melatonin (N-acetyl-5-methoxytryptamine) [8]. Figure 1 shows the melatonin and 3-indoleacetic acid (IAA) biosynthetic pathway. The red arrows represent the melatonin synthesis pathway, the green arrows are the IAA synthesis pathway, and the blue arrow identifies the synthesis pathway shared by both molecules. Both melatonin and IAA are synthesized from tryptophan. Tryptophan is converted into 5-hydroxytryptophan and tryptamine before the pathway branches into IAA biosynthesis. Tryptamine and 5-hydroxytryptophan are then synthesized serotonin and melatonin. Biosynthetic enzymes are denoted as follows: TDC, tryptophan decarboxylase; T5H, tryptophan 5-hydroxylase; SNAT, serotonin N-acetyltransferase; HIOMT, hydroxyindole-O-methyltransferase.

Melatonin is biosynthesized in most plant tissues [8,9,10]. The biosynthesis of melatonin in plants has been the subject of several recent studies. Byeon et al. (2014) demonstrated the presence of melatonin in rice plants and showed that rice contains genes that encode all the enzymes responsible for the production of melatonin [8]. While the presence of melatonin in plants has been established, the specific details of its biosynthesis remain unclear. The biosynthesis of melatonin in plants may follow a similar pathway to its synthesis in animals, involving tryptophan as the precursor molecule [11]. However, the specifics of the melatonin synthetic route require further study.

## 3. Effects of Melatonin on Plant Growth

Several studies have shown that melatonin can regulate plant physiological functions; it generally promotes root, shoot, and explant growth [12,13]. Hernández-Ruiz et al. provided compelling evidence for the direct involvement of melatonin in the promotion of plant organ growth. Specifically, melatonin was found to increase the length of the roots and coleoptiles of monocots and exhibit a high relative auxinic concentration (10%–55%) [13].

Melatonin is closely related to the growth and developmental status of plants, and its content varies greatly during different growth periods. Melatonin concentrations were found to be relatively low (nearly 15 ng/g FW) in the first and third stages of sweet cherry fruit development, but substantially higher (reaching a peak value of 35.6 ng/g FW) in the second stage [14]. In this stage, the cells elongate significantly, cell volume expands, the embryo develops, and seeds germinate more rapidly. In addition, RT-PCR experiments revealed that the PaTDC gene, which encodes the rate-limiting enzyme in melatonin synthesis, has a higher expression level of this gene during the second phase than during the first and last stages. Therefore, melatonin is likely involved in and contributes to fruit development processes. In addition to this, there is much evidence that there can be differences in melatonin content. The melatonin content of the crocus orchid increases in photoperiodic growth, and if the plant is transferred to a dark environment, the melatonin content decreases significantly, which is the effect brought by light and the environment. Melatonin content is also higher in some grass crops, such as rice and maize, and lower in tomatoes, bananas, and beet at the pg/g FW level [15], which is determined by different varieties. However, these phenomena and phenotypes require further study to reveal the mechanisms by which melatonin affects crop development.

In addition to the positive effects, we should also be concerned about the negative effects of melatonin. Relatively high levels of melatonin in plants lead to the production of RNS and ROS, which in turn play an important role in plant tolerance to unfavorable environmental conditions [16]. The inability of the plant’s detoxification system to fully degrade certain foreign compounds leads to increased levels of phytotoxicity [17]. Melatonin has ushered in a new era of plant adaptation mechanisms. Therefore, the study of exogenous melatonin in stressed plants from the perspective of plant adaptation and survival has received extensive attention from researchers. Another study revealed the mechanism of melatonin signaling during maize growth from the perspective of glucose metabolism, demonstrating that 10 μM melatonin promoted increases in leaf length and the ratio of root length to root weight, whereas 100 μM of melatonin resulted in growth inhibition [18]. At a low concentration (10 μM), melatonin stimulates the export of triose phosphate (a raw material for sucrose synthesis) from the chloroplast to the cytoplasm by upregulating the activity of the Calvin cycle enzymes. Experimental results indicated that the enzymatic activity related to sucrose synthesis in the source was upregulated, resulting in the accumulation of a large amount of sucrose which could be either intracellularly stored or transported to other sites for glycolysis to release energy or for storage. In contrast, sucrose metabolism is not affected by high melatonin levels. However, there was a significant downregulation of enzyme indicators related to photosynthesis and starch metabolism in the source, which decreased the amount of triose entering the cytosol, along with a decrease in sucrose content. Additionally, hexokinase was shown to be downregulated in response to high concentrations of melatonin, hindering plant utilization of sucrose in a glycolytic manner. This made us realize the two sides of melatonin in plants. In summary, at the physiological level of growth, very high concentrations of melatonin had notably different effects from those of low concentrations of melatonin, mainly through the inhibition of the Calvin cycle, starch anabolism, glycolysis-related enzyme activities, and expression levels.

The role of melatonin as a regulator of circadian rhythms has been clearly demonstrated in mammals [19,20]. In plants, circadian oscillators are able to regulate the phases of a variety of biological processes, such as gene and metabolic regulation and protein stability [21]. The circadian rhythm of melatonin levels observed in unicellular algae and higher plants suggests that melatonin plays a role in regulating photoperiodic and rhythmic phenomena, i.e., melatonin has similar functions in plants and animals [22]. In experiments with *Chenopodium rubrum*, changes in melatonin content brought about by light versus darkness were observed, which showed a clear cyclic pattern [23]; in experiments with water hyacinth, the highest melatonin levels were found in plants grown under natural conditions, with a peak in the late light phase of the light–dark cycle [24]. In any case, there appears to be a circadian rhythm of melatonin in plants.

## 4. Effects of Melatonin on Plant Reproduction Material

The general method of preserving tree species ex vivo is cryopreservation. However, plant somatic cells can suffer from water stress, desiccation, and cryoinjury during cryopreservation, resulting in a variety of growth problems during the process of redifferentiation after the thawing of cryopreserved materials. It was discovered that treating immature shoots with 0.1–0.5 µM of melatonin in preculture and a redifferentiation medium for 24 h resulted in a considerable increase in their redifferentiation ability compared to that of untreated nodes. Similarly, the redifferentiation ability of salidroside-pre-frozen calli increased following a 0.1 µM melatonin treatment [25].

Polyethylene glycol (PEG) can be used to simulate drought stress by controlling the water potential (ψw) [26]. When cucumber seeds were germinated in an 18% PEG + 100 µM melatonin solution, the seed germination rate was approximately 7% higher than that in the 18% PEG + water group, demonstrating that melatonin might alleviate water stress and promote seed germination [27]. In addition, melatonin (1 µM) improved cucumber reproduction and increased germination under salt stress via regulating abscisic acid (ABA) and gibberellin (GA) synthesis [28]. When applied exogenously, melatonin was shown to boost the tolerance of the grape root system to water stress, thereby encouraging growth. Melatonin was also found to maintain the normal structure of chloroplast internal lamellar systems and prevent ultrastructural damage caused by drought stress [29]. Melatonin treatment frequently increases seed germination to 2–3 times that of untreated seeds [30]. Although melatonin has been repeatedly shown to promote seed germination, the mechanism underlying this effect is still unclear.

## 5. Response of Melatonin to Plant Stresses

### 5.1. Plant Disease Resistance

Plant diseases lead to the greatest productivity and economic losses in agriculture worldwide, and numerous organizations have developed various techniques to manage plant diseases. Exogenous administration of melatonin (0.05–0.5 mM) modifies the activity of antioxidant and plant defense-related enzymes and enhances resistance to apple blotch, one of the most serious plant diseases [31]. Furthermore, Ishihara et al. (2008) discovered that activating the tryptophan synthesis pathway led to an increase in serotonin levels within rice leaves. This increase in serotonin ultimately inhibited the development of fungal hyphae within rice leaf tissues, resulting in a highly efficient defense mechanism [32]. Although the authors did not mention melatonin, this possibility cannot be ruled out, because serotonin is a biosynthetic precursor of melatonin [7,9,13,33]. In another study, researchers applied 10 µM melatonin to reduce the expression of genes associated with disease in Arabidopsis, such as PR1, PDF1.2, and ICS1 [34]. This provides evidence that melatonin may act as a signaling molecule that helps plants resist infections.

### 5.2. Phytoharmful Chemical Stress Pathways

One of the biochemical indicators of senescence is chlorophyll degradation and loss, and the positive effects of melatonin on this process have been reported [35]. According to one study, melatonin delays drought- and dark-induced leaf senescence in apples by preserving photosystem II performance and suppressing chlorophyll content loss under stress conditions. Melatonin was found to lower chlorophyll enzyme gene expression in Arabidopsis leaves treated with paraquat (PQ), indicating that chlorophyllase is involved in the light-regulated process of chlorophyll breakdown [36]. In addition, a transcriptome analysis revealed the upregulation of many genes involved in pathways of stress-related phytohormones (e.g., jasmonic and abscisic acids), which may also indicate that melatonin elicits multiple stress responses in plants [37].

Szafrańska et al. (2017) also found that the levels of chlorophyll A and B, as well as and carotenes (CARs) were higher after than before a melatonin treatment, which may confirm that melatonin positively affects the biosynthesis of chlorophyll and CARs [36]. After a PQ treatment for 24 h, the pheophytin content in the leaf discs, which is related to photosynthesis, significantly increased in the control group (to over 0.4 mg/g FW), whereas only a slight increase was observed in the melatonin-treated group. These findings show the beneficial effect of melatonin in slowing down chlorophyll decomposition via the formation of pheophytins under PQ-induced oxidative stress. Taken together, these studies showed that melatonin protects the chlorophyll content of pea leaves during PQ-induced oxidative stress by delaying chlorophyll degradation and accelerating its de novo synthesis.

### 5.3. High-Nitrate-Stress Pathways

Nitrogen is a key element in the synthesis of nucleic acid molecules, proteins, chlorophyll, multiple hormones, and secondary metabolites [38]. Consequently, nitrogen assimilation efficiency and availability significantly influence plant growth, development, and metabolism [39]. Most plants obtain inorganic nitrogen from nitrate and ammonia nitrogen [40]. Excess nitrate–nitrogen increases ion toxicity and osmotic, secondary, and ionic stress; reduces photosynthesis and enzyme activity; and decreases crop output quantity and quality. When nitrogen intake exceeds the plant digestion capability, large levels of ammonium and nitrate nitrogen accumulate in the plant, causing plant salt toxicity and even secondary soil salinization, all of which harm plant growth [41].

In a recent study, researchers divided alfalfa seedlings into three groups of nine seedlings: the control group (CK), the high nitrate group (HN), and the high nitrate + melatonin group (HN + MT). They measured several phenotypic parameters of seedlings in these three groups [42]. Their findings indicated that melatonin improved alfalfa growth and development under high nitrate stress. Melatonin participates in free radical scavenging activities to protect the cells from the harmful effects of severe nitrate stress. The direct antioxidant and free radical scavenging properties of melatonin are largely due to its electron-rich aromatic indole ring, which makes it a potent electron donor that significantly reduces oxidative stress [43,44]. Furthermore, under nitrate stress, melatonin directly increased the activities of glutamine synthetase (GS), glutamate synthase (GOGAT), and other enzymes. Proline is a multifunctional chemical that helps plants improve and enhance their drought resistance. It accumulates during stress and is destroyed to supply energy to support plant growth after the stress is relieved [45]. When melatonin relieved high-nitrate stress, the proline levels increased again.

Adenosine triphosphate (ATP) is a crucial signaling molecule in cell communication and directly supplies energy for cell metabolism, gene expression, and other energy-consuming pathways in all living organisms [46,47,48]. Under high nitrate stress, alfalfa consumed more adenosine triphosphate and generated adenosine diphosphate it also consumed and generated more adenosine monophosphate than the control. Melatonin increased the adenosine triphosphate re-synthesis pathway, protecting alfalfa from high nitrate concentrations, which provide insight into the mechanisms that plants use to respond to stress and to maintain their metabolic activities under adverse environmental conditions.

Overall, melatonin plays a crucial role in mitigating the negative impact of high nitrate stress on plant physiology. By enhancing the activity and expression of nitrogen-metabolizing enzymes, melatonin effectively curbs the production of harmful nitrate–nitrogen and ammonia–nitrogen in alfalfa. Moreover, the hormone helps to maintain the energy homeostasis of the plant and safeguards it from nitrate-induced damage.

### 5.4. Drought Stress

**Figure 2:**
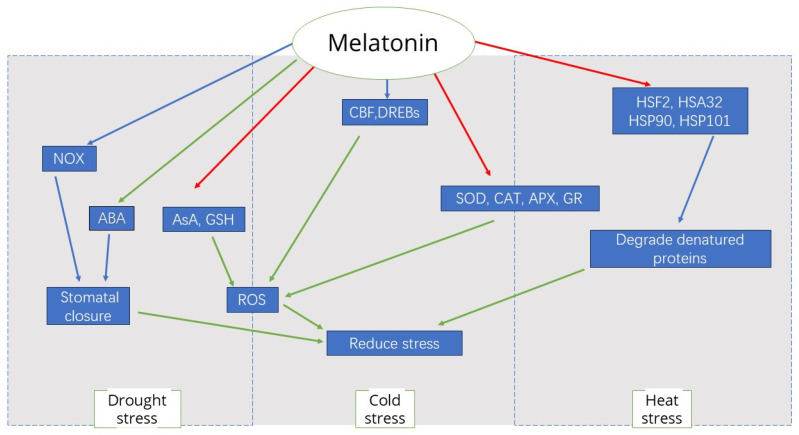
Melatonin has a protective effect on plants in abiotic stress environments, and the regulatory pathways associated with melatonin under three stress environments (drought, cold, and heat) are illustrated in Figure 2. Red arrows represent promotion and upregulation, while green arrows indicate inhibition and downregulation. In drought stress, melatonin alters stomatal conductance by affecting NOX and ABA synthesis catabolism. Under cold and heat stress, melatonin protects cells by inducing upregulation of transcription factors to reduce oxidative damage. Influential factors on the dashed boxes indicate pathways that are present in both stresses.

In recent years, there have been many cases of growth inhibition or direct death of trees, which are inextricably linked to drought stress and to some extent indicate that drought stress is one of the major abiotic stresses limiting the normal growth and development of trees. Exogenous application of melatonin as a way of alleviating drought stress has been well studied. The most important reason for melatonin-mediated improvement of photosynthesis under drought stress is that melatonin protects the chloroplast structure in leaves from oxidative damage, which is progressively shortened under water-deficit conditions, accompanied by disruption of membranes, stroma lamellae, grana and thylakoids [29]. However, melatonin treatment prevented all these adverse effects of drought on chloroplast structure. In addition, the restoration of stomatal length and the shape of fenestrated tissues, as well as the reduction of cell damage in spongy tissues, contributed to the improvement of photosynthesis in drought-stressed plants after melatonin treatment [29]. In the Apple genus, it has been illustrated that plant melatonin improves the condition of plant stomata under drought conditions, resulting in longer and wider stomata and larger pore size [49]. The increase in stomatal conductance contributes to better movement of water and carbon dioxide, which ultimately favors photosynthesis in melatonin-treated plants (Figure 2) [50].

### 5.5. Cold and Heat Temperature Stress

Melatonin has been shown to significantly alleviate cold stress in many plants and to have a protective effect on cold tissues. Cold treatment can affect endogenous melatonin concentrations in plants, and a paper using bermudagrass (*Cynodon dactylon* (L). Pers.) also noted that cold treatment caused a significant increase in endogenous melatonin to 3-fold higher than normal treatment [51]. Lupine plants grown at 6 °C showed a 2.5-fold increase in melatonin content compared to control plants grown at 24 °C [52]. Exogenous treatments of various plants with melatonin, such as such as rice, tomato, Bermudagrass, and Lupinus albus, were confirmed to reverse the inhibitory effects of low temperature on germination and root growth [53]. Exogenous application of melatonin to cold-stressed wheat increased SOD, CAT, APX, and GR activities, which reduced ROS levels and caused less oxidative damage to improve plant growth (Figure 2) [54]. Treatment of Arabidopsis with melatonin upregulated the expression of genes encoding C-repeat binding factors (*CBFs*)/drought-responsive element-binding factors (*DREBs*); the cold-responsive gene *COR15a*; *CAMTA1*, encoding a transcription factor involved in freezing and drought stress tolerance; and *ZAT10* and *ZAT12*, two important transcriptional activators encoding ROS-related antioxidant genes [53,55,56,57,58]. These data suggest that melatonin is associated with the upregulation of specific cold-responsive genes, supporting the hypothesis that melatonin has a protective effect against abiotic stresses.

Under conditions of heat stress, genes responsible for melatonin biosynthesis are often activated, leading to higher levels of melatonin. For example, melatonin levels are increased in rice [59], and elevated temperatures increase melatonin levels in green micro-algae Ulva sp. [22]. Heat stress (37 °C) resulted in a 2- to 5-fold increase in endogenous melatonin levels in Arabidopsis seedlings. Exogenous melatonin treatment increased the survival of heat-stressed Arabidopsis compared to non-melatonin-treated plants [60]. A study reported that melatonin application activated stress-responsive genes in Bermuda grass. The C-repeat binding factor/dehydration-responsive element-binding protein (*CBF/DREB*) genes and target genes, heat shock transcription factors (TFs), zinc finger TFs, *WRKY, MYB, bHLH* genes, and hormone-related genes showed 16-fold higher expression than in control plants [51]. Heat shock factors are the main mediators of melatonin-mediated heat stress response. Heat shock factors (HSFA2 and HSA32) and heat shock proteins (HSP90 and HSP101) have been demonstrated that contribute to melatonin-mediated heat tolerance in Arabidopsis [61]. Melatonin protects cellular proteins in tomato by inducing *HSPs* and autophagy to remodel or degrade denatured proteins under heat stress (Figure 2) [62]. Exogenous melatonin increased the activity of enzymes related to nitrogen metabolism, increased nitrate content, limited ammonium content at high temperatures, and increased the resistance of cucumber seedlings to heat stress [14]. Melatonin application has the potential to reverse the inhibitory effects of light and high temperature on photosensitive and heat-sensitive *Phacelia tanacetifolia* Benth seeds [63].

## 6. Coordination of Carbon and Nitrogen Metabolism

Nitrogen is a significant element found in living organisms; it cannot be used directly but permeates cells in the form of ammonium ions through various nitrogenous molecules (mostly proteins and nucleic acids). It is absorbed by plants as nitrate or ammonium ions via the root system [64,65]. Nitrates, being the principal nitrogen source for plant species, are first converted into nitrite by nitrate reductase (NR) in the cytosol before being transformed into ammonium by nitrite reductase (NiR). The ammonium ion rapidly combines with ketoglutarate to generate glutamine via the combined activity of glutamine synthetase (GS), glutamate oxoglutarate transaminase (GOGAT), and glutamate dehydrogenase (GDH) [66]. Exogenous melatonin significantly increased the expression of key genes of nitrogen metabolism and the activities of key enzymes GOGAT, NR, GS and GDH. In studies on soybean, it was hypothesized that exogenous melatonin could increase the activity of key enzymes of nitrogen metabolism to some extent, reducing NO_3_^−^ to NH_4_^+^, thereby increasing the rate of the GS/GOGAT cycle and improving the overall nitrogen metabolism of soybean [67]. Intermediate metabolites in these pathways are required for plant growth because they serve as precursors in the synthesis of amino acids and almost all nitrogenous molecules. Photosynthesis and mitochondria provide ATP, reducing agents, and carbon skeletons for nitrogen absorption and amino acid biosynthesis [64,68]. Consequently, it is vital for plant development that the balance between nitrogen and carbon metabolism is maintained.

In one study on maize seedling growth, researchers sprayed an equal number of maize seedlings with 10, 100, and 1000 μM of melatonin and showed that melatonin administration to seedlings considerably increased root length, plant height, leaf area, soluble carbohydrate and protein content, and total chlorophyll content [69]. Melatonin was shown to increase the rates of biosynthetic processes and stimulate plant growth.

Molecular-level studies have demonstrated that melatonin addition enhanced the activities of nitrate reductase, glutamate synthetase, nitrite reductase, and glutamine synthetase, as well as gene expression. The limiting enzyme in nitrogen assimilation is nitrate reductase, which catalyzes the reduction of nitrate to nitrite. Increased activity of this enzyme helps prevent excess nitrate buildup by coordinating the carbon and nitrogen metabolism, whereas nitrite reductase further reduces nitrite to ammonium, and low levels of ammonium favor the carbon and nitrogen metabolism [14,70,71]. Studies have revealed that melatonin increased plant nitrate levels and that the conversion of nitrate to nitrite was significantly accelerated because of the increased nitrate reductase activity, which was also upregulated, catalyzing the conversion of nitrite to ammonium, which decreased the nitrite concentration. Melatonin was eventually found to improve plant usage of nitrate nitrogen by enhancing the activity of enzymes involved in nitrogen assimilation.

In summary, the link between carbon and nitrogen metabolism is strong, and melatonin has made significant contributions to the coordinated management of carbon and nitrogen metabolism.

## 7. Melatonin Interacts with Other Phytohormones

Melatonin acts synergistically or antagonistically with multiple phytohormones to regulate plant growth and development. For instance, when the plant body receives a certain intensity of melatonin signal, it can activate the auxin synthesis pathway, exhibiting a synergistic effect with auxin. Under stress conditions such as drought and unsuitable temperature, however, melatonin inhibits the expression of auxin synthesis-related enzymes [72,73]. This results in a reduction in metabolic levels, inhibition of plant growth, and improved plant resistance.

Melatonin has been shown to be particularly effective in mitigating the effects of drought stress. Abscisic acid (ABA) is one of the most important hormones that is affected by drought stress, and exogenous melatonin is capable of inhibiting abscisic acid synthesis and reducing ABA levels during drought. The NCED3 gene, which encodes an epoxycarotenoid oxygenase, is a key enzyme in the ABA synthesis pathway. Studies have shown that NCED3 expression is upregulated under drought conditions in various plants, leading to the synthesis of large amounts of ABA and exacerbation of plant senescence [74]. Melatonin works to downregulate NCED3 expression, activate genes of ABA catabolic enzymes such as CYP707, reduce ABA levels in plants, and delay plant senescence under adverse stresses [75,76].

Together, melatonin interacts with other phytohormones (e.g., IAA, GA) to regulate plant growth and development, and it is particularly effective in mitigating the effects of drought stress by suppressing ABA synthesis and delaying plant senescence. As such, melatonin may hold significant potential for improving crop yields and enhancing plant resistance to environmental stresses.

## 8. Signaling Pathways Upstream of Melatonin in Plants

Cand2 (also known as GPCR) plays an essential role as a melatonin receptor in Arabidopsis. Recently, studies have demonstrated that melatonin interacts with Cand2 receptors situated on the plasma membrane to cause stomatal closure in Arabidopsis. This closure occurs via two essential signaling pathways, the ROS and Ca^2+^ signaling pathways. However, when Cand2 knockout mutants are studied, it is observed that this process does not take place [4]. ROS signaling regulates several cellular pathways; for example, it can boost plant resilience to disease and promote plant development by modulating polyamine metabolic pathways [77]. This research might provide new insights into how melatonin improves immunological function and promotes plant development.

**Figure 3:**
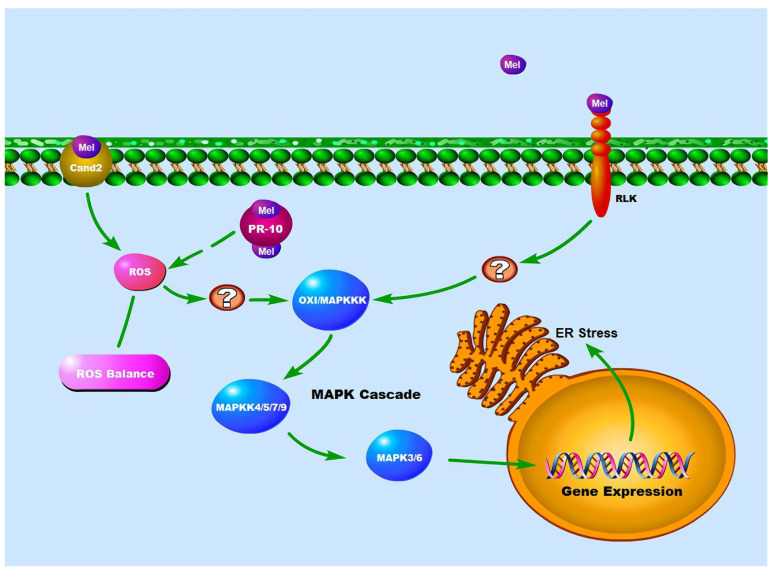
Signaling pathways of melatonin. Mel: melatonin; RLK: receptor like kinases; Cand2: melatonin receptor in Arabidopsis; ROS: reactive oxygen species; ROS balance: reactive oxygen species balance; PR-10: pathogenesis-related proteins; MAPK: mitogen-activated protein kinase;”?”: unknown.

The Cand2 receptors also stimulate the mitogen-activated protein kinase (MAPK) signaling cascade, either directly or indirectly. Melatonin-mediated MAPK cascade pathways have been investigated under two stress conditions: pathogen infection and endoplasmic reticulum stress [78,79]. When Arabidopsis was challenged by pathogens, melatonin production transiently enhanced, which activates MAPKKKs, including OXI1, resulting in the activation of different genes via the MAPK4/5/7/9 and MAPK3/6 signaling cascades (Figure 3). MAPK3/6 double mutants exhibit no melatonin-mediated pathogen resistance. However, SNAT1 knockout mutants show considerably decreased MAPK3/6 activation, leading to enhanced pathogen susceptibility [78,79]. Melatonin was also linked to the activation of the endoplasmic reticulum (ER) resident chaperones and ER stress [80]. Melatonin-mediated stress resistance was eliminated in MAPK3/6 knockout mutants, whereas ER stress-induced ER chaperone gene expression decreased in SNAT1 knockout mutants, demonstrating that the MAPK cascade is involved in melatonin-mediated ER stress tolerance.

The widespread receptor-like kinases (RLKs) on the plasma membrane may act as another melatonin receptor in Arabidopsis [78]. RLKs are a class of transmembrane receptor proteins whose structure includes a transmembrane region, a cytoplasmic kinase domain, and an extracellular signaling molecule binding domain [81]. Studies have shown that the major RLK in Arabidopsis is the FLS2 receptor. There is evidence that the FLS2 receptor stimulates the expression of several defense-related genes, including glutathione S-transferase 1 (GST1), PR1, and PR5, through activating the MAPK cascade and WRKY transcription factors via FLG22 binding [82]. However, whether the signaling transduction after melatonin binding to FLS2 also occurs via the FLS2 pathway and the detailed process of gene expression activation are still under investigation.

Pathogenesis-related proteins (PR-10 proteins) are a group of proteins that are biosynthesized in plants upon exposure to various stresses, including pathogen attack, abiotic stress, and hormonal signals. PR-10 proteins are known to possess antimicrobial activity and have been extensively studied for their role in plant defense mechanisms [83]. However, recent studies have suggested that PR-10 proteins may also play a key role in plant physiology through their interaction with melatonin [84,85]. The binding of melatonin to PR-10 proteins has been shown to have a correlation with reactive oxygen species (ROS) levels in plant cells. The molecular mechanisms involved in the modification period after PR-10 binds melatonin to ROS levels are still being explored. It has been suggested that this interaction may modulate the activity of enzymes involved in ROS metabolism, leading to changes in ROS levels in plant cells and thus activation of the MAPK pathway [86]. Further research is needed to fully elucidate the molecular mechanisms involved in this process and to determine the physiological significance of this interaction in plant growth and development.

To date, research on plant melatonin receptors remains relatively limited, particularly with regard to signal transduction pathways downstream of melatonin. The controversies and lack of exploration regarding these pathways have become an interesting subject in the fields of plant cytology and physiological biochemistry. It is expected that future studies will focus on elucidating more details about the molecular mechanisms and signaling pathways involved in melatonin’s effects on plant growth and development.

## 9. Discussion

In this review, the focus is on the diverse roles of melatonin, which is a pleiotropic, amphipathic molecule and a chemical indoleamine in nature. The exact details of its biosynthesis remain unclear and may follow a pathway similar to its synthesis in animals, involving tryptophan as a precursor molecule. However, the specifics of the melatonin synthesis route need to be further investigated. Then, new questions are brought up, for example, how does the amount of tryptophan in plants relate to the amount of melatonin? Does the melatonin concentration in the plant organism increase after treatment with tryptophan? These questions have not yet been answered.

This paper highlights melatonin’s significance in regulating glucose metabolism, nitrogen metabolism, carbon and nitrogen metabolism coordination, and external stress resistance in plants. Additionally, the effects of exogenous melatonin application on downstream metabolic pathways in plant cells are discussed. The latest advances in understanding the signal transduction pathway upstream of melatonin are also reported. It is worth noting that melatonin plays an important role in various physiological processes of plant growth and development, as well as in stress response.

Since the discovery of plant melatonin receptors and the proposal of signal transduction pathways, the physiological and biochemical effects of melatonin in plants have been extensively researched. Like other phytohormones, melatonin has multifaceted functions in plant physiology. It plays a crucial role in regulating plant growth and development by promoting cell division and elongation, stimulating the production of plant hormones such as auxins, and inhibiting abscisic acid synthesis. These hormones are involved in various plant processes, including seed germination, leaf and stem growth, and stress tolerance. Additionally, melatonin is essential for plant biochemistry and physiology, particularly in the context of field crops. Research has shown that melatonin may contribute to crop productivity and nutritional value and help address global food security issues.

Although current studies have preliminarily revealed the synthesis, signal transduction pathways, and major physiological functions of melatonin in plants, a clear picture of melatonin signal transduction; the tissue specificity of melatonin synthesis; its response; and how melatonin regulates gene expression remain to be investigated. In 1977, the birth of next-generation sequencing technology (Sanger sequencing) marked the possibility of gene sequencing, which progressed from the end feet method, to microarray chips, to the development of higher-throughput next-generation sequencing (NGS) technologies such as next-generation sequencing and three-generation sequencing. The advantage of three-generation sequencing technology in directly sequencing RNA not only facilitates transcriptome research but also facilitates the development of novel research methods such as single-cell sequencing (scRNA-seq) and spatial transcriptome. To date, Hi-C sequencing technology, developed based on “circularization enhancement”, and spatial enhanced resolution omics sequencing (Stereo-seq) technology enables ultra-high precision dissection of genes and cell-changing processes during development in both temporal and spatial dimensions [87,88]. Currently, deconvolution algorithms that have rarely been reported in plant research can be used to estimate the cellular composition of a tissue by using bulk RNA-seq data, which will alleviate the cost and improve the efficiency of single-cell analysis [89]. The methods above will be important tools for studying alterations in the genomic structure and function of plant cells under the actions of melatonin, as well as the functions of melatonin synthesis and response to changes in gene expression and the molecular functions of the expressed products, so as to construct a precise spatiotemporal picture of the processes involved in the synthesis and regulation of melatonin in plants.

In this comprehensive review, we delve into the latest research on the contributions of melatonin to plant stress resistance and disease prevention. The research has shown that melatonin, at specific concentrations, can improve the activities of enzymes involved in the carbon–nitrogen metabolism pathway. Additionally, it can inhibit disease-related gene expression and promote chlorophyll stability, thereby improving plants’ resistance to adverse stresses. We must note, however, that there is still a notable dearth of studies on the role of melatonin in controlling plant microbial and insect-related diseases. Future research will undoubtedly focus on identifying the precise function of melatonin in assisting disease and pest control. Through such investigations, we anticipate the development of novel plant growth-regulating products incorporating melatonin, which can be commercialized to control crop growth and enhance yields. Thus, the applications of melatonin may play an instrumental part in promoting sustainable agriculture practices, facilitating the growth and maturation of crops, and sustaining the environment.

## Data Availability

Not applicable.

## References

[1] Lerner A.B., Case J.D., Takahashi Y., Lee T.H., Mori W. (1958). Isolation of melatonin, a pineal factor that lightens melanocytes. J. Am. Chem. Soc..

[2] Van Tassel D.L., Roberts N.J., O’Neill S.D. (1995). Melatonin from higher plants: Isolation and identification of N-acetyl-5-methoxytryptamine. Plant Physiol..

[3] Reiter R.J., Tan D.X., Zhou Z., Cruz M.H.C., Fuentes-Broto L., Galano A. (2015). Phytomelatonin: Assisting plants to survive and thrive. Molecules.

[4] Wei J., Li D.X., Zhang J.R., Shan C., Rengel Z., Song Z.B., Chen Q. (2018). Phytomelatonin receptor PMTR1-mediated signaling regulates stomatal closure in *Arabidopsis thaliana*. J. Pineal Res..

[5] Maronde E., Stehle J. (2007). The mammalian pineal gland: Known facts, unknown facets. Trends Endocrinol. Metabol..

[6] Hardeland R., Madrid J.A., Tan D.X., Reiter R.J. (2012). Melatonin, the circadian multioscillator system and health: The need for detailed analysis of peripheral melatonin signal. J. Pineal Res..

[7] Falcón J., Besseau L., Fuentès M., Sauzet S., Magnanou E., Boeuf G. (2009). Structural and functional evolution of the pineal melatonin system in vertebrates. Ann. N. Y. Acad. Sci..

[8] Byeon Y., Lee H.Y., Lee K., Park S., Back K. (2014). Cellular localization and kinetics of the rice melatonin biosynthetic enzymes SNAT and ASMT. J. Pineal Res..

[9] Park S., Lee K., Kim Y.S., Back K. (2012). Tryptamine 5-hydroxylase-deficient Sekiguchi rice induces synthesis of 5-hydroxytryptophan and N-acetyltryptamine but decreases melatonin biosynthesis during senescence process of detached leaves. J. Pineal Res..

[10] Arnao M.B., Hernandez-Ruiz J. (2014). Melatonin: Plant growth regulator and/or biostimulator during stress?. Trends Plant Sci..

[11] Byeon Y., Tan D.X., Reiter R.J., Back K. (2015). Predominance of 2-hydroxymelatonin over melatonin in plants. J. Pineal Res..

[12] Murch S., Campbell S.S.B., Saxena P.K. (2001). The role of serotonin and melatonin in plant morphogenesis. Regulation of auxin-induced root organogenesis in in vitro-cultured explants of *Hypericum perforatum* L.. In Vitro Cell. Dev. Biol. Plant..

[13] Hernández-Ruiz J., Cano A., Arnao M.B. (2005). Melatonin acts as a growth-stimulating compound in some monocot species. J. Pineal Res..

[14] Zhao N., Sun Y., Wang D.Y., Zheng J. (2012). Effects of exogenous melatonin on nitrogen metabolism in cucumber seedlings under high temperature stress. Plant Physiol. J..

[15] Arnao M.B. (2014). Phytomelatonin: Discovery, Content, and Role in Plants. Adv. Bot..

[16] Tan D.X., Hardeland R., Manchester L.C., Korkmaz A., Ma S., Rosales-Corral S., Reiter R.J. (2012). Functional Roles of Melatonin in Plants, and Perspectives in Nutritional and Agricultural Science. J. Exp. Bot..

[17] Dixit P., Mukherjee P.K., Sherkhane P.D., Kale S.P., Eapen S. (2011). Enhanced Tolerance and Remediation of Anthracene by Transgenic Tobacco Plants Expressing a Fungal Glutathione Transferase Gene. J. Hazard. Mater..

[18] Zhao H., Su T., Huo L., Wei H., Jiang Y., Xu L., Ma F. (2015). Unveiling the mechanism of melatonin impacts on maize seedling growth: Sugar metabolism as a case. J. Pineal Res..

[19] Reiter R.J. (1993). The melatonin rhythm: Both a clock and a calendar. Experientia.

[20] Reppert S.M., Weaver D.R. (2002). Coordination of circadian timing in mammals. Nature.

[21] Arnao M.B., Hernández-Ruiz J. (2015). Functions of melatonin in plants: A review. J. Pineal Res..

[22] Tal O., Haim A., Harel O., Gerchman Y. (2011). Melatonin as an antioxidant and its semi-lunar rhythm in green macroalga *Ulva* sp.. J. Exp. Bot..

[23] Kolář J., Johnson C.H., Macháčková I. (2003). Exogenously applied melatonin (N-acetyl-5-methoxytryptamine) affects flowering of the short-day plant Chenopodium rubrum. Physiol. Plant.

[24] Tan D., Reiter R.J., Manchester L.C., Yan M., El-Sawi M., Sainz R.M., Mayo J.C., Kohen R., Allegra M., Hardeland R. (2002). Chemical and physical properties and potential mechanisms: Melatonin as a broad spectrum antioxidant and free radical scavenger. Curr. Top. Med. Chem..

[25] Zhao Y., Qi L.W., Wang W.M., Saxena P.K., Liu C.Z. (2011). Melatonin improves the survival of cryopreserved callus of *Rhodiola crenulata*. J. Pineal Res..

[26] Nepomuceno A., Oosterhuis D., Stewart J. (1998). Physiological responses of cotton leaves and roots to water deficit induced by polyethylene glycol. Environ. Exp. Bot..

[27] Zhang N., Zhao B., Zhang H.J., Weeda S., Yang C., Yang Z.C., Ren S., Guo Y.D. (2013). Melatonin promotes water-stress tolerance, lateral root formation, and seed germination in cucumber (*Cucumis sativus* L.). J. Pineal Res..

[28] Zhang H.J., Zhang N., Yang R.C., Wang L., Sun Q.Q., Li D.B., Cao Y.Y., Weeda S., Zhao B., Ren S. (2014). Melatonin promotes seed germination under high salinity by regulating antioxidant systems, ABA and GA4 interaction in cucumber (*Cucumis sativus*). J. Pineal Res..

[29] Jiang F.M., Teng F.X., Zhi Z.W., Yu L.F., Zhu M.X., Zhen W.Z. (2014). The ameliorative effects of exogenous melatonin on grape cuttings under water-deficient stress: Antioxidant metabolites, leaf anatomy, and chloroplast morphology. J. Pineal Res..

[30] Aguilera Y., Herrera T., Benítez V., Arribas S.M., de Pablo A.L.L., Esteban R.M., Martín-Cabrejas M.A. (2015). Estimation of scavenging capacity of melatonin and otherantioxidants: Contribution and evaluation in germinated seeds. Food Chem..

[31] Yin L., Wang P., Li M., Ke X., Li C., Liang D., Wu S., Ma X., Li C., Zou Y. (2013). Exogenous melatonin improves *Malus* resistance to Marssonina apple blotch. J. Pineal Res..

[32] Ishihara A., Hashimoto Y., Tanaka C., Dubouzet J.G., Nakao T., Matsuda F., Nishioka T., Miyagawa H., Wakasa K. (2008). The tryptophan pathway is involved in the defense responses of rice against pathogenic infection via serotonin production. Plant J..

[33] Reiter R.J. (1991). Melatonin synthesis: Multiplicity of regulation. Adv. Exp. Med. Biol..

[34] Lee H.Y., Byeon Y., Back K. (2014). Melatonin as a signal molecule triggering defense responses against pathogen attack in Arabidopsis and tobacco. J. Pineal Res..

[35] Wang P., Sun X., Li C.H., Wei Z., Liang D., Ma F. (2013). Long-term exogenous application of melatonin delays drought-induced leaf senescence in apple. J. Pineal Res..

[36] Katarzyna S., Russel J.R., Malgorzata M.P. (2017). Melatonin Improves the Photosynthetic Apparatus in Pea Leaves Stressed by Paraquat via Chlorophyll Breakdown Regulation and Its Accelerated de novo Synthesis. Front. Plant Sci..

[37] Weeda S., Zhang N., Zhao X., Ndip G., Guo Y., Buck G.A., Fu C., Ren S. (2014). Arabidopsis transcriptome analysis reveals key roles of melatonin in plant defense systems. PLoS ONE.

[38] Wen B., Xiao W., Mu Q., Li D., Chen X., Wu H., Li L., Peng F. (2020). How does nitrate regulate plant senescence?. Plant Physiol. Biochem..

[39] Hachiya T., Sakakibara H. (2017). Interactions between nitrate and ammonium in their uptake, allocation, assimilation, and in plants. J. Exp. Bot..

[40] Zhu Y., Huang X., Hao Y., Su W., Liu H., Sun G., Chen R., Song S. (2020). Ammonium transporter (BcAMT1.2) mediates the interaction of ammonium and nitrate in *Brassica campestris*. Front. Plant Sci..

[41] Hao F., Liu X., Fan J. (2017). Study on key enzyme activity in nitrogen metabolism and the content of molybdenum and iron in alfalfa under different NO3--N/NH4+-N ratio. Agr. Res. Arid Areas.

[42] Zhao C., Cao X., Niu J. (2021). Effects of Melatonin on Morphological Characteristics, Mineral Nutrition, Nitrogen Metabolism, and Energy Status in Alfalfa Under High-Nitrate Stress. Front. Plant Sci..

[43] Tan D.X., Manchester L.C., Esteban-Zubero E., Zhou Z., Reiter R.J. (2015). Melatonin as a Potent and Inducible Endogenous Antioxidant: Synthesis and Metabolism. Molecules.

[44] Martinez G.R., Almeida E.A., Klitzke C.F., Onuki J., Prado F.M., Medeiros M.H., Mascio P.D. (2005). Measurement of Melatonin and Its Metabolites: Importance for the Evaluation of Their Biological Roles. Endocrine.

[45] Hayat S., Hayat Q., Alyemeni M.N., Wani A.S., Pichtel J., Ahmad A. (2012). Role of proline under changing environments: A review. Plant Signal. Behav..

[46] Cao Y., Tanaka K., Nguyen C.T., Stacey G. (2014). Extracellular ATP is a central signaling molecule in plant stress responses. Curr. Opin. Plant. Biol..

[47] De Col V., Fuchs P., Nietzel T., Elsässer M., Voon C.P., Candeo A., Seeliger I., Fricker M.D., Grefen C., Møller I.M. (2017). ATP sensing in living plant cells reveals tissue gradients and stress dynamics ofenergy physiology. eLife.

[48] Tripathi D., Zhang T., Koo A.J., Stacey G., Tanaka K. (2018). Extracellular ATP acts on jasmonate signaling to reinforce plant defense. Plant Physiol..

[49] Tao W., Ma Y., Zhou Y., Li L., Xu D., Luo S., Zhuang W., Zhang W., Xie Y. (2023). Physiological and proteomic analyses of Malus crabapples exposed to long-term warming and short-term heat shock treatments reveal the response characteristics of photosynthetic apparatus. Sci. Hortic..

[50] Cui G., Zhao X., Liu S., Sun F., Zhang C., Xi Y. (2017). Beneficial Effects of Melatonin in Overcoming Drought Stress in Wheat Seedlings. Plant Physiol. Biochem..

[51] Shi H., Jiang C., Ye T., Tan D.X., Reiter R.J., Zhang H., Liu R., Chan Z. (2015). Comparative Physiological, Metabolomic, and Transcriptomic Analyses Reveal Mechanisms of Improved Abiotic Stress Resistance in Bermudagrass (*Cynodon dactylon* (L.) Pers.) by Exogenous Melatonin. J. Exp. Bot..

[52] Arnao M.B., Hernández-Ruiz J. (2013). Growth Conditions Determine Different Melatonin Levels in *Lupinus albus* L.. J. Pineal Res..

[53] Wang P., Sun X., Wang N., Tan D.X., Ma F. (2015). Melatonin Enhances the Occurrence of Autophagy Induced by Oxidative Stress in Arabidopsis Seedlings. J. Pineal Res..

[54] Turk H., Erdal S., Genisel M., Atici O., Demir Y., Yanmis D. (2014). The Regulatory Effect of Melatonin on Physiological, Biochemical, and Molecular Parameters in Cold-Stressed Wheat Seedlings. Plant Growth Regul..

[55] Bajwa V.S., Shukla M.R., Sherif S.M., Murch S.J., Saxena P.K. (2014). Role of Melatonin in Alleviating Cold Stress in *Arabidopsis thaliana*. J. Pineal Res..

[56] Shi H., Chan Z. (2014). The Cysteine2/Histidine2-Type Transcription Factor ZINC FINGER OF ARABIDOPSIS THALIANA 6-Activated C-REPEAT-BINDING FACTOR Pathway Is Essential for Melatonin-Mediated Freezing Stress Resistance in Arabidopsis. J. Pineal Res..

[57] Zhao D., Yu Y., Shen Y., Liu Q., Zhao Z., Sharma R., Reiter R.J. (2019). Melatonin Synthesis and Function: Evolutionary History in Animals and Plants. Front. Endocrinol..

[58] Gu Q., Chen Z., Yu X., Cui W., Pan J., Zhao G., Xu S., Wang R., Shen W. (2017). Melatonin Confers Plant Tolerance Against Cadmium Stress via the Decrease of Cadmium Accumulation and Reestablishment of MicroRNA-Mediated Redox Homeostasis. Plant Sci..

[59] Byeon Y., Back K. (2014). Melatonin Synthesis in Rice Seedlings In Vivo Is Enhanced at High Temperatures and Under Dark Conditions Due to Increased Serotonin N-Acetyltransferase and N-Acetylserotonin Methyltransferase Activities. J. Pineal Res..

[60] Hernández I.G., Gomez F.J.V., Cerutti S., Arana M.V., Silva M.F. (2015). Melatonin in Arabidopsis thaliana Acts as Plant Growth Regulator at Low Concentrations and Preserves Seed Viability at High Concentrations. Plant Physiol. Biochem..

[61] Shi H., Tan D.X., Reiter R.J., Ye T., Yang F., Chan Z. (2015). Melatonin Induces Class A1 Heat-Shock Factors (HSFA 1s) and Their Possible Involvement in Thermotolerance in Arabidopsis. J. Pineal Res..

[62] Xu W., Cai S.Y., Zhang Y., Wang Y., Ahammed G.J., Xia X.J., Shi K., Zhou Y.H., Yu J.Q., Reiter R.J. (2016). Melatonin Enhances Thermotolerance by Promoting Cellular Protein Protection in Tomato Plants. J. Pineal Res..

[63] Tiryaki I., Keles H. (2012). Reversal of the Inhibitory Effect of Light and High Temperature on Germination of *Phacelia tanacetifolia* Seeds by Melatonin. J. Pineal Res..

[64] Erdal S., Turk H. (2016). Cysteine-induced upregulation of nitrogen metabolism-related genes and enzyme activities enhance tolerance of maize seedlings to cadmium stress. Environ. Exp. Bot..

[65] Duan W., Wang Q., Zhang H., Xie B., Li A., Hou F., Dong S., Wang B., Qin Z., Zhang L. (2018). Comparative study on carbon–nitrogen metabolism and endogenous hormone contents in normal and overgrown sweetpotato. S. Afr. J. Bot..

[66] Jabeen N., Ahmad R. (2017). Growth response and nitrogen metabolism of sunflower (*Helianthus annuus* L.) to vermicompost and biogas slurry under salinity stress. J. Plant Nutr..

[67] Cao L., Qin B., Gong Z., Zhang Y. (2022). Melatonin Improves Nitrogen Metabolism During Grain Filling Under Drought Stress. Physiol. Mol. Biol. Plants.

[68] Lin Y., Zhang J., Gao W., Chen Y., Li H., Lawlor D.W., Paul M.J., Pan W. (2017). Exogenous trehalose improves growth under limiting nitrogen through upregulation of nitrogen metabolism. BMC Plant Biol..

[69] Serkan E. (2019). Melatonin promotes plant growth by maintaining integration and coordination between carbon and nitrogen metabolisms. Plant Cell Rep..

[70] Zhang R.M., Sun Y.K., Liu Z.Y., Jin W., Sun Y. (2017). Effects of melatonin on seedling growth, mineral nutrition, and nitrogen metabolism in cucumber under nitrate stress. J. Pineal Res..

[71] Liang B., Ma C., Zhang Z., Wei Z., Gao T., Zhao Q., Ma F., Li C. (2018). Long-term exogenous application of melatonin improves nutrient uptake fuxes in apple plants under moderate drought stress. Environ. Exp. Bot..

[72] Arnao M.B., Hernández-Ruiz J. (2019). Melatonin: A new plant hormone and/or a plant master regulator?. Trends Plant Sci..

[73] Arnao M.B., Hernández-Ruiz J. (2021). Melatonin against environmental plant stressors: A review. Curr. Protein Pept. Sci..

[74] Lee S.U., Mun B.G., Bae E.K., Kim J.Y., Kim H.H., Shahid M., Choi Y.I., Hussain A., Yun B.W. (2021). Drought Stress-Mediated Transcriptome Profile Reveals NCED as a Key Player Modulating Drought Tolerance in *Populus davidiana*. Front. Plant Sci..

[75] Arnao M.B., Hernández-Ruiz J. (2018). Melatonin and its relationship to plant hormones. Ann. Bot..

[76] Nakashima K., Yamaguchi-Shinozaki K. (2013). ABA signaling in stressresponse and seed development. Plant Cell Rep..

[77] Léo G., Caroline B., Stéphane G. (2021). Polyamines: Double agents in disease and plant immunity. Trends Plant Sci..

[78] Lee H.Y., Back K. (2016). Mitogen-activated protein kinase pathways are required for melatonin-mediated defense responses in plants. J. Pineal Res..

[79] Lee H.Y., Back K. (2017). Melatonin is required for H_2_O_2_-and NO-mediated defense signaling through MAPKKK3 and OXI1 in Arabidopsis thaliana. J. Pineal Res..

[80] Lee H.Y., Back K. (2018). Melatonin plays a pivotal role in conferring tolerance against endoplasmic reticulum stress via mitogen-activated protein kinases and bZIP60 in Arabidopsis thaliana. Melatonin Res..

[81] Shiu S.H., Karlowski W.M., Pan R., Tzeng Y.H., Mayer K.F., Li W.H. (2004). Comparative analysis of the receptor-like kinase family in Arabidopsis and rice. Plant Cell.

[82] Asal T., Tena G., Plotnikova J., Willmann M.R., Chiu W.L., Gomez-Gomez L., Boller T., Ausubel F.M., Sheen J. (2002). MAP kinase signaling cascade in Arabidopsis innate immunity. Nature.

[83] Fernandes H., Michalska K., Sikorski M., Jaskolski M. (2013). Structural and functional aspects of PR-10 proteins. FEBS J..

[84] Sliwiak J., Dauter Z., Jaskolski M. (2016). Crystal structure of Hyp-1, a Hypericum perforatum PR-10 protein, in complex with melatonin. Front. Plant Sci..

[85] Sliwiak J., Sikorski M., Jaskolski M. (2018). PR-10 proteins as potential mediators of melatonin-cytokinin cross-talk in plants: Crystallographic studies of LIPR-10.2B isoform from yellow lupine. FEBS J..

[86] Feng K., Lu J., Chen Y., Luo Y., Hu Y., Li X., Zhong S., Cheng L. (2022). The coordinated alterations in antioxidative enzymes, *PeCu/ZnSOD* and *PeAPX2* expression facilitated in vitro *Populus euphratica* resistance to salinity stress. Plant Cell Tissue Organ Cult..

[87] Rao S.S., Huntley M.H., Durand N.C., Stamenova E.K., Bochkov I.D., Robinson J.T., Sanborn A.L., Machol I., Omer A.D., Lander E.S. (2014). A 3D map of the human genome at kilobase resolution reveals principles of chromatin looping. Cell.

[88] Xia K., Sun H.X., Li J., Li J., Zhao Y., Chen L., Qin C., Chen R., Chen Z., Liu G. (2022). The single-cell stereo-seq reveals region-specific cell subtypes and transcriptome profiling in Arabidopsis leaves. Dev. Cell.

[89] Sutton G.J., Poppe D., Simmons R.K., Walsh K., Nawaz U., Lister R., Gagnon-Bartsch J.A., Voineagu I. (2022). Comprehensive evaluation of deconvolution methods for human brain gene expression. Nat. Commun..

